# Effects of Ochratoxin A on Livestock Production

**DOI:** 10.3390/toxins2071796

**Published:** 2010-07-08

**Authors:** Gianni Battacone, Anna Nudda, Giuseppe Pulina

**Affiliations:** Dipartimento di Scienze Zootecniche, Università degli Studi di Sassari, Via De Nicola 9, 07100 Sassari, Italy; Email: anudda@uniss.it (A.N.); gpulina@uniss.it (G.P.)

**Keywords:** Ochratoxin A, livestock performance, livestock health, food safety

## Abstract

Ochratoxin A (OTA) contamination often causes large economic losses on livestock production. The intake of feed contaminated by OTA also represents a potential risk for animal health and a food safety issue due to the transfer of the toxin through the food chain to humans. The aim of this paper is to review the available literature on: (1) the frequency and degree of occurrence of OTA in different feedstuffs; (2) the toxicological effects of OTA intake on the performance of the main livestock (*i.e.*, poultry, swine, cattle, goats and sheep); and (3) the transfer of OTA, or its metabolites, from animal feed into animal products such as milk, meat and eggs.

## 1. Introduction

Ochratoxins are worldwide spread secondary metabolites synthesized mainly by some toxigenic species of *Aspergillus* and *Penicillium* [1] that contaminate various raw agricultural commodities and have dangerous effects on animals and humans. The chemical structure of ochratoxin A (OTA), the most common ochratoxin, consists of a dihydroisocoumarin part coupled, via its 7-carboxy group, with a L-beta-phenylalanine part.

The most relevant toxic effect of OTA in animal cells is the inhibition of protein synthesis, but other important effects are lipid peroxidation, DNA damage and oxidoreductive stress [2]. Mutagenic effects in mammalian cells, as a consequence of DNA damage induction, have been reported [3]. Further evidence suggests a genotoxic carcinogen role of OTA, due to the induction of oxidative DNA lesions combined with the creation of covalent bond between OTA reactive metabolites and the damaged DNA [4]. OTA effects of on animal health are strongly influenced by its kinetic (*i.e.*, toxicokinetic and toxicodinamic) parameters, which differ between ruminant and monogastric species. In monogastric (e.g., pigs and poultry species), OTA is absorbed from the gastrointestinal tract without, or with little, prior degradation. On the contrary, in ruminants OTA is subjected to microbial degradation in the rumen before any essential uptake. The most relevant microbial degradation of OTA is carried out by enzymes able of peptide hydrolysis, yielding phenylalanine and the nontoxic dihydroisocoumarin part, currently named as Ochratoxin-alpha [5]. In the bloodstream, almost all OTA is strongly bound to serum proteins, mainly albumin. Interaction between OTA and serum albumin affects the half-life of the toxin in the bloodstream and differs substantially between species [6]. Sreemannarayana *et al.* [7] reported an OTA serum half-life of about 77 h in pre-ruminant calves, whereas Galtier *et al.* [6] reported a half-life of 88.8, 8.2 and 4.1 h in pigs, rabbits and chickens, respectively. Actually, differences in OTA serum half-life account for most of the high variability of OTA toxic effect observed among animal species.

Considering the risk of potential transfer from feeds to animal tissues and products, legislative authorities have enacted or proposed regulations for controlling OTA in foods and/or feed commodities. In 2006, the Commission of the European Communities [8] recommended the member States to ensure the respect of the guidance values indicated for the acceptability of composed feeds, cereal and cereal products for animal feeding. Values vary between feedstuffs and animal species (Table 1). 

**Table 1:** Guidance values for OTA in feedingstuffs with a moisture content of 12%, as set in the Commission Recommendation 2006/576/EC.

**Products Intended for Animal Feed**	**Guidance Valuein mg/kg**
Feed materials	
Cereals and cereal products	0.25
Complementary and complete feedingstuffs	
Complementary and complete feedingstuffs for pigs	0.05
Complementary and complete feedingstuffs for poultry	0.1

Nevertheless, when cereals and cereal products are fed directly, the daily amount of OTA ingested should not be higher than the OTA intake by animal for which only complete feedstuffs are used in the diet. So far the U.S. Food and Drug Administration [9] has not set advisory limits or action levels for OTA in any commodity, even if this institution has included OTA in the list of Potentially Hazardous Contaminants in Animal Feed and Feed Ingredients. Most information available on the toxic effects of OTA on animals was obtained in experiments carried out on laboratory animals. Nevertheless, in the late 1960s, the scientific community began to study the adverse effects of the ingestion of feed contaminated by *Aspergillus ochraceus* on livestock species [10]. 

Several ochratoxigenic mycobiota can grow in grains and forages used for animal feeding and produce OTA and other mycotoxins. Thus, farm animals may be exposed to the toxic effects of OTA together with those of other toxins. Most common mycotoxins occurring together with OTA are: citrinin, zearalenone, penicillic acid and aflatoxin B1 [11]. *In vivo* experiments carried out to evaluate effects of a mycotoxin combination on animals usually yield results that are very difficult to interpret. To overcome these difficulties, Speijers and Speijers [11] stressed the importance of understanding the way mycotoxins, such as OTA, can interact at the cellular level with other fungal compounds. A special care is needed in the theoretical assumptions, in the planning of experiments and in the statistical analysis of results.

Negative economic implications of OTA on livestock have been reported since the early 1970s [12]. Because ruminant species are commonly considered to be less susceptible to OTA effects, most research has focused on monogastric species.

Even though the toxic effects of OTA on animals differs markedly between species, studies carried out on monogastrics represent an interesting model for approaching the study of OTA effects on human health. This is a relevant point, considering that OTA is suspected of being the main etiological agent responsible for human Balkan endemic nephropathy and associated urinary tract tumors [4].

Hereinafter, most relevant information about the occurrence of OTA contamination in feedstuffs and the effects of OTA on performances and OTA transfer into animal products of the main livestock species is given briefly.

## 2. Occurrence of OTA in Feeds

Prevention of pre-harvest and post-harvest natural contamination of feedstuffs by OTA is a basic tool in the strategy to minimize the subsequent occurrence of OTA into the feed and food chain.

It is well-known that cereals, mainly in Northern America, Northern and Western Europe and other temperate regions, may be exposed to colonization by fungi of the genus *Penicillium* and *Aspergillus* able to produce OTA. These molds do not invade the crop in the field but mainly in the post-harvest phase [1].

However, mold species producing OTA differ among ecological conditions and commodities that characterize different geographical regions. In general, *Penicillium verrucosum* is responsible of OTA contamination in cool-temperate conditions, whereas *Aspergillus ochraceus* is probably the main ochratoxigenic species in hot-tropical regions [13].

Because the main abiotic factors which influence mold growth and OTA production are water availability and temperature, Magan and Aldred [1] suggested that cereals should be quickly dried, reaching a moisture content lower than 14.5% and maintaining this condition during storage, to avoid OTA contamination.

In their review, Scudamore *et al.* [13] reported that OTA is mainly concentrated in the seed coat of cereals, which is often used for animal feeding. Moreover, on-farm production and storage of barley and wheat with a high moisture content increases the risk of mold growth and toxin production. Magan and Aldred [1] suggested the following moisture content values during storage as a safe threshold: 14–14.5% for wheat, barley and oats; 14% for maize; 13–14% for rice and 7–8% for rape seed.

Cairns-Fuller *et al.* [14] reported that water activity, temperature and CO_2_ concentration are main factors affecting *P. verrucosum* growth and OTA production in wheat grain produced and stored in cooler Northern European climates. Moreover, authors reported that at least 50% of CO_2_ is required to inhibit *P. verrucosum* growth and to reduce by more than 75% its OTA production in wheat grain with a water activity within the interval 0.90–0.995. Therefore, a moisture content of 17–18% (water activity of 0.80–0.83) can be considered as a threshold for avoiding any potential growth of mold and OTA production in wheat grain.

Levels of OTA of 110 to 150 ppb have been found by Shotwell *et al.* [15] in ground corn suspect for the presence of OTA. Moreover, they claimed that until 1969 there has been not any report for the presence of OTA as a natural contaminant even though this mold was widely distributed in nature. Since then, there have been several investigations on the occurrence of mycotoxins, particularly OTA, in feeds and commodities used for animal nutrition.

The fact that the contamination of cereals, or derived products, by OTA is a worldwide problem is confirmed by several studies carried out during the last four decades in several geographic and climatic conditions.

In a survey on over 500 samples of home-grown cereals (barley, wheat and oats) in England and Wales from 1976 to 1979, OTA was found as the most frequent mycotoxin (12.8% of samples being positive) [16] .

Scudamore *et al.* [17], in an investigation carried out on more than 350 samples of feeds collected in the United Kingdom (limit of quantification of the analysis, LOQ = 0.001 mg/kg), reported that barley and wheat were the feed ingredients with the highest occurrence of OTA (60 and 40% of positive samples, respectively), even if seven out of 15 samples of dried peas/beans also contained OTA.

In samples of several cereals and other feeds, such as soybean and sunflower, collected in Hungary, Rafai *et al.* [18] reported that the average concentration of OTA of soybean, maize and rye was 350, 320 and 250 ppb. Average contamination in other feeds, such as wheat, barley, oat, triticale and sunflower, was lower than 200 ppb. Such contamination levels in Hungary were confirmed by the results of Fazekas *et al.* [19] that reported contamination frequencies of 35.0, 15.6, and 26.7% for barley, wheat and maize, respectively. Moreover, OTA average concentrations were 72 ppb in barley (range: from 0.14 to 212 ppb), 12.2 ppb in wheat (range: from 0.3 to 62.8 ppb) and 4.9 ppb in maize (range: from 1.9 to 8.3 ppb).

Czerwiecki *et al.* [20,21] reported OTA occurrence in cereal grains from conventional and ecological farms in Poland during 1997 and 1998. In 1997, the frequency and the concentration of OTA were higher in rye, barley and wheat grains from ecological farms (range 0.2–57 ppb) than in conventional cultivation. Differently in 1998, the frequency of OTA contamination of rye and barley was similar for conventional and ecological farms, even if the overall range of OTA concentration in those cereals was higher in ecological (from 1.4 to 35.3 ppb) than in conventional (from 8.8 to 9.7 ppb) farms. Moreover, in that year the frequency of contaminated samples was 48% and 23% in cereals of conventional and ecological farms, respectively. The overall range of OTA concentration in wheat grain varied greatly between conventional (from 0.6 to 1024 ppb) and ecological farm (from 0.8 to 1.6 ppb). Those results suggest that contamination frequency may differ greatly across years and farming systems and that the latter are not a factor able to control the extent of grain contamination by OTA.

Garaleviciene *et al.* [22] showed an OTA content greater than 10 ppb in about 92% of samples of wheat, barley, oats and 52 mixed feeds collected in Lithuanian farms. The magnitude of OTA contamination in cereals produced and stored in Baltic regions was confirmed by Baliukoniene *et al.* [23], who analyzed OTA concentration in wheat and barley produced and stored under different conditions in Lithuania. OTA concentrations were 3.19, 1.78 and 1.13 ppb in wheat grains from small, medium and large granaries, respectively, whereas it varied between 0.92 and 0.37 ppb in barley samples.

In 96 samples of poultry feed collected in Brazil, Rosa *et al.* [24] reported an occurrence of OTA in 100% of the feeds, with concentrations varying from 1.3 to 80 ppb. Moreover, 84 out of 340 *Aspergillus* and *Penicillium* strains isolated were able to produce OTA under *in vitro* conditions. Successively, Fraga *et al.* [25] studied the mycological contamination, potential mycotoxin production and occurrence of OTA in 144 samples of poultry feeds and feed ingredients collected from a factory of Rio de Janeiro State in Brazil from April 2003 to March 2004. The study showed that 100% of the poultry feed samples were contaminated with OTA, at levels varying between 0.017 and 0.197 mg/kg, with a mean value of 0.098 mg/kg. Mycological analysis indicated that OTA contamination could be due to the presence of two strains of OTA-producing *Aspergillus melleus*.

In a survey on feeds used for swine nutrition in Brazil, Rosa *et al.* [26] reported that OTA was detected in all 144 feeds analyzed. The concentration of OTA ranged from 42 and 224 ppb in corn samples, from 36 to 120 ppb in brewers grains samples and from 28 to 135 ppb in finished feed samples.

In a recent investigation conducted by Binder *et al.* [27], ingredients and feeds were sampled in animal farms in European, Asian and Pacific regions from October 2003 to December 2005. The occurrence of OTA in Asia and in the Pacific region was detected (limit of detection, LOD = 0.002 mg/kg) in 0.26 of the complete feeds and 0.25 of maize. The incidence of OTA in feeds sampled in Europe and others Mediterranean region was 0.42 in wheat, 0.73 in complete feeds and 0.68 in other feed ingredients.

The risk of intake of relevant amounts of OTA is much lower in cattle than in pigs and poultry species, because cattle feeding is mostly based on forages and only partially on cereals, which are the feeds with the highest risk of contamination.

In a survey on 290 samples of grass silage, whole-crop maize silage and whole-crop wheat or triticale silage collected in randomly selected Dutch dairy farms from 2002 to 2004, Driehuis *et al.* [28] reported that none of the silages contained OTA (LOQ = 8 ppb). In another investigation analyzing the OTA content (LOQ = 8 ppb) in silages and concentrates used in the diet of lactating cows in 24 dairy farms across the Netherlands, Driehuis *et al.* [29]did not detect OTA in any of the feeds samples.

Lund and Frisvad [30] suggested that an infestation of *Penicillium verrucosum* higher than 7% in cereals indicates OTA contamination. Subsequently, Lindblad *et al.* [31] developed a logistic model able to predict the probability of reaching OTA levels exceeding various thresholds in cereal grains on the basis of storage conditions (temperature and water activity) and number of colonies of *P. verrucosum*. Because mycological analyses are generally less expensive than chemical analysis to determine mycotoxins, the use of fungal concentrations for estimating the OTA level may be a cheap tool for managing the risk of OTA contamination in cereal grains,

These monitoring techniques, apart from being of great importance for controlling contamination risk in grains used in animal feeding, may be particularly helpful to reduce the exposure to OTA intake in food human consumption.

The Joint FAO/WHO Expert Committee on Food Additives [32] has further emphasized the relevance of OTA in human food mainly due to consumption of contaminated foodstuffs such as cereal grains. Considering the global importance of cereals in the human/animal diet and their susceptibility to attack by molds producing OTA, Duarte *et al.* [33] showed in their review an updated anthology of data on OTA as a worldwide contaminant in cereal crops and cereal based products destined to human consumption. 

## 3. Effects of OTA on Livestock Performance and Health

As stated in the previous section, the toxicity of OTA varies between animal species [2]. In the following section, the main effects of OTA on swine, poultry, cattle, sheep and goat are reviewed. 

### 3.1. Swine

**Figure 1:**
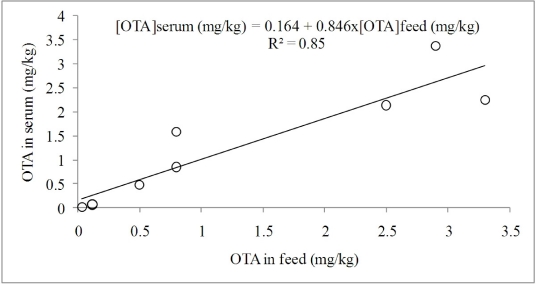
Relationship between the concentration of OTA in the diet and its concentration in pig blood serum.

Economic losses of swine farms that can be ascribed to OTA feed contamination mainly consists of negative effects on pig’s health and productive performance, but also to the cost for the disposal of carcasses that cannot be sold. Residual OTA may be found in animal tissues such as serum, kidney, liver and muscle. Since the 1920s, a morphologically characterized nephropathy has been described in pigs of Northern Europe countries [34]. Elling and Moller [35] suggested that OTA contamination of moldy barley grains fed to pigs was responsible for progressive interstitial fibrosis and regressive tubular changes with thickening of the basement membranes in their kidneys. According to the authors, the intake of feeds contaminated by OTA was the probable cause of a disease named Denmark nephropathy in pigs. In the study of Szczech *et al.* [36], young swine fed rice culture contaminated by OTA (0.2 to 0.6 mg/kg body weight of OTA daily) or pure toxin (2.0 or 1.0 mg/kg body weight daily) had abrupt depression and reduction in feed intake, with consequent body weight loss, followed by dehydration, diarrhea, polyuria and polydipsia. The animals given pure OTA were dead or moribund in five to six days. In a trial carried out in two Swedish pig herds, Rutqvist *et al.* [37] found relationships between several degrees of nephropathy and OTA content in organs such as kidneys, liver, whole blood and plasma. Authors suggested the OTA content in blood, where highest values were found, as a possible indicator for clinical investigations of ochratoxicosis in live pigs or for the evaluation of feed OTA contamination. Moreover, in a subsequent study, the same research group [38] reported that the analysis of a single blood sample from a herd was able to give a reliable estimation of OTA feed content. On the other hand, strong correlations between feed OTA content and its residue in kidney [37], liver and adipose tissue [39], and muscle [40], indicated that OTA concentration in feed could be used to predict its residue in pig tissues and organs. Relationships between average OTA concentration in serum and its correspondent concentration in the diet fed to pigs (varying from 0.0322 to 3.3 mg/kg), observed in several studies [41,42,43,44,45], are depicted in Figure 1. Considering that OTA content in blood serum reaches a plateau after 10-13 days [46], data reported in Figure 1 were generated in experiments where exposure to OTA lasted at least 14 days. The obtained regression equation confirms the close and positive relationship between OTA content in the feed and its residue in blood serum. Aoudia *et al.* [45] proposed to convert the concentration of OTA in serum to its concentration in the blood, considering that about 57% of the blood volume of pig is due to plasma.

During the last decades, studies carried out in several countries have reported the frequency and degree of occurrence of OTA in the blood and many tissues of slaughtered pigs. Frohlich *et al.* [47], in a survey on pigs of the Canadian market, reported that frequency of occurrence of OTA in serum was highest in summer whereas its concentration value was highest in autumn. Authors explained these results with seasonal differences in temperatures and duration of grain storage, which affect growth of molds and OTA production.

Aoudia *et al.* [45] reported following regression equations between OTA in kidney and liver (ppb) and its plasma concentration (ppb) in piglets fed OTA contaminated diets at 117.45 ± 4.74 and 118.13 ± 2.85 ppb after four weeks:

[OTA] kidney = 1.169 + 0.132 × [OTA] plasma (R^2^ = 0.81; n = 19; P < 0.05)

[OTA] liver = 0.128 + 0.014 × [OTA] plasma  (R^2^ = 0.61; n = 21; P < 0.05)

Regression coefficients indicated that the residue of OTA was higher in kidney than in liver.

Apart from the accumulation of OTA residue in kidney and liver tissues, the ingestion of feed contaminated by OTA induced an increase in weight of these organs that are fundamental for the detoxification or elimination processes [42,45]. However, such effects were observed in experiments in which the level of OTA contamination was higher than the recommended guidance value of 0.05 mg/kg indicated by the European Commission [8] for pig feeds. In heavy pigs fed a diet with 0.05 mg/kg of OTA for four weeks, Battacone *et al.* [48] did not observe any effects of the toxin on weight daily gain, hematological parameters and kidney and liver integrity and functionality.

A depressive effect of OTA on pig appetite was reported in some experiments [41,49] but not in others [43,45]. These contrasting results could be related to differences in experimental conditions such as the level and type of feed contamination (natural or artificial) and the length of animal exposure to the toxin.

Reduction in weight gain caused by OTA contamination was observed in experiments in which the feed intake was depressed [41] or not [43]. On the other hand, Aoudia *et al.* [45] did not find any statistical difference in weight gain between piglets fed a diet with OTA at 0.117 mg/kg for four weeks and piglets fed a control diet (OTA at 0.85 ppb).

**Figure 2:**
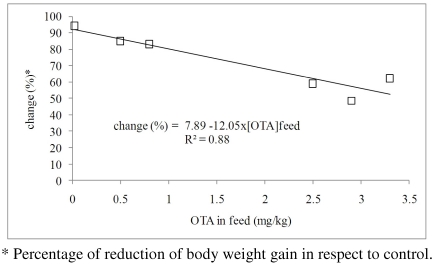
Relationship between the concentration of OTA in the diet and change in body weight gain, as % of relative control.

Data from experiments [41,42,43], in which a reduction of weight gain caused by the OTA feed contamination was observed, are plotted in Figure 2. Though only limited data, a significant decrease of daily weight gain for increasing OTA concentrations in feed can be observed. The equation slope indicates an average reduction of 12% in weight gain of pigs for an increase of 1 mg/kg of OTA in the diet.

A reduction of feed efficiency (gain of body weight per amount of feed intake) by OTA was observed in an experiment in which feed intake was not affected by the toxin [43] and also in a trial in which feed intake by pigs fed diets contaminated by OTA was lower than in a control group [41].

All above mentioned results suggest that the negative effects of OTA on pig growth can be the due to a reduction of feed intake or to a direct toxic effect of OTA on animals, or both. Moreover, alteration of metabolism, as revealed by several anomalous serum blood parameters such as hyperproteinemia and azotemia, hypocholesterolemia and hypercalcemia [41], may have a role.

Because OTA is a well-known potent inhibitor of protein synthesis, Birò *et al.* [50] investigated the effects of OTA on serum and seminal plasma OTA concentration, spermatological parameters, and histological evaluation of testes and epididymis on male pigs. Treated boars (average body weight of 250 kg) were fed OTA at a daily dose of 0.08 mg/kg, in addition to the toxin content of the control diet, for six weeks, followed by a nine-week withdrawal period. OTA concentration in serum reached its maximum at about one week from the start of toxin administration, whereas its concentration in seminal plasma remained considerably lower and reached the highest concentration four weeks later than in serum. The volume of the ejaculate was not significantly affected by OTA, whereas the viability, longevity and motility of spermatozoa were negatively affected in samples of semen re-evaluated 24 h after collection. Histological parameters such as number, morphology and quantitative disproportion of germ cells typical of different developmental stages were not affected by OTA. However, the authors concluded that further research was needed to investigate reasons for the small proportion of morphologically disturbed epithelial cells in the tubular lumina in tests of boars fed artificially contaminated diet.

#### 3.1.1. Interaction of OTA with Other Mycotoxins

Since many fungi can grow and produce their mycotoxins in similar environmental conditions, OTA rarely occurs as a single contaminant in feedstuffs [51]. Moreover, it is a common practice in swine farms to blend several grain sources. The intake of diet contaminated by OTA and other mycotoxins may lead to interactive toxic effects on animals [52], depending on the absorption rates of the different mycotoxins in their gastrointestinal tract. Unfortunately, studies about the effects of the combined exposure to mycotoxins on animal performance are very complicated because it is difficult to establish antagonistic, additive or synergistic effects through *in vivo* experiments. A possible approach to understand interactions between OTA and other mycotoxins in animals is the development of simulation models able to mimic toxicokinetic behavior, metabolism and toxicodynamic aspects of toxins in animals. Recently, Avvantaggiato *et al.* [53] developed a dynamic multicompartmental model to simulate simultaneous intestinal absorption of OTA and aflatoxin B1 from artificially contaminated corn in the upper gastrointestinal tract of healthy pigs. Results confirmed the usefulness of these tools in increasing the knowledge of the kinetic of OTA in the gastrointestinal tract of pigs. The implementation of theoretical models could also be a valuable tool to support the interpretation of data from *in vivo* experiments. 

#### 3.1.2. OTA Binders Supplements

Various feed supplements have been tested to verify their protective effects against the harmful effects of OTA on pigs. Sequestering agents have been added to contaminated feed, aiming to reduce the adsorption of the toxin by the gastrointestinal tract and thus decrease the effect of mycotoxins on pig health. In particular, several binders have been tested for their effectiveness in detoxifying pig feeds contaminated by OTA. The use of binders in the livestock industry must be preceded with *in vivo* or *in vitro* experimental evaluation of the product. Jarczyk *et al.* [44] tested the effectiveness of activated charcoal and aluminosilicate in deactivating the OTA at 0.032 mg/kg in pig feed. Both binders, added at the doses recommended by the producers, did not affect the OTA serum concentration. A summary of the effectiveness of different binders in counteracting the negative effects of OTA in pigs, and other animal species, is reported in the review of Huwig *et al.* [54]. The most satisfying results against the adverse effects of OTA in pigs were obtained by adding activated charcoal, whereas no significant effects were observed by adding bentonite or hydrated sodium calcium aluminosilicates [54].

Another way of reducing the bioavailability of OTA in the gastrointestinal tract of pigs was tested by Aoudia *et al.* [45].The authors showed that the addition of 1% of micronized wheat fibers to piglet diets contaminated by OTA at about 118 ppb reduced its concentration in plasma and in other tissues such as kidney and liver. Moreover, the micronized fibers significantly prevented the increase of the weight of organs involved in detoxifying or eliminating OTA, such as kidneys and liver.

### 3.2. Poultry

Toxic effects of OTA on poultry have been reported since the 1960s [55]. Burditt *et al.* [56] suggested that the response to ocratoxicosis differed between poultry species. In their review, Marquardt and Frohlich [57] reported that the first documented field outbreak of ochratoxicosis in turkeys, laying hens and broiler chickens was described by Hamilton *et al.* [58]. The main toxic effects observed in turkeys were high mortality, nephrotoxicity, and reduced feed intake. A reduction in growth rate and feed efficiency, occurrence of nephropathy and of air sacculitis were reported for broiler chickens. Nephropathy associated with reduction of egg production and quality were observed in laying hens.

Poultry are less sensitive to OTA than swine, probably due to their higher capacity of excreting OTA compared to other species [6]. Nevertheless, several experiments reported negative effects of OTA feed contamination on poultry performances. 

Stoev *et al.* [59] observed macroscopical, histopathological, hematological and biochemical changes in chicks exposed to a diet contaminated with OTA at 5 mg/kg for six and 10 weeks. The blood level of chicks showed an alteration of hematopoiesis characterized by a slight anaemia. Moreover, increased serum levels of uric acid, urea and creatinine, and reduced levels of serum total protein were observed, together with a high urinary protein excretion, indicating a possible severe damage to kidney functions. All these metabolic disorders may be responsible for the high weight observed for the liver and kidneys; organs involved in the detoxification and elimination of OTA from blood. On the other hand, some lymphoid organs as thymus, bursa of Fabricius and spleen showed degenerations and decreased weight caused by the reduced number of antibody-producing cells. 

In a necropsy analysis of chickens fed a diet containing OTA at 2 mg/kg for three and four weeks, Santin *et al.* [60] found alterations in internal organs such as pale swollen kidneys and enlarged, yellowish and friable at palpation livers. In addition, histopathological examinations showed vacuolation and megalocytosis of hepatocytes, hyperplasia of the biliary epithelium and hypertrophy of renal proximal tubular epithelial cells. In broiler chicks fed a diet with 2 mg/kg of OTA, no changes were observed within the first three weeks of treatment [61]. However, after five weeks, remarkable changes in organs such as swelling of kidneys, slight enlargement of liver and reduction in size of the bursa of Fabricius. Moreover, histopathological analyses showed marked changes in the lesion score of organs, which was the highest for kidneys followed by the liver, bursa, spleen and thymus. Kumar *et al.* [62] confirmed that OTA is more a nephrotoxin than a hepatotoxin for broilers: most relevant effects observed on animals fed a diet contaminated with OTA at 2 mg/kg were atrophy of the bursa, thymus and spleen along with the depletion of lymphocytes. In a recent study carried out on two commercial poultry farms, Bozzo *et al.* [63] detected OTA in all feed samples, with a concentration ranging between 0.160 and 0.332 mg/kg. The OTA contaminated feed was administered to animals for at least two months. Postmortem inspection and the cytological and histological examinations of the layer hens evidenced gross and microscopical lesions in the kidneys and liver. Sawale *et al.* [64] reported negative effects of OTA on performance, hematobiochemical disturbances and severe immunosupression in laying hens fed a diet contaminated with OTA at 1mg/kg of feed for 60 days.

Several experiments were carried out in order to evaluate the effect of OTA feed contamination on broiler weight gain [59,65,66,67,68,69,70]. Results are summarized in Table 2. The reduction in growth performance of broilers is positively related with the concentrations of OTA in feed and with the length of exposure to the toxin.

**Table 2:** Magnitude of the negative effects of OTA on body weight gain (BW), as percentage of control, of broilers fed diets with different toxin contents and times of exposure.

Breed	Initial age	OTA in feed (mg/kg)	Exposition time (weeks)	Change in BW, as % of control	Reference
commercial broilers	1-day-old	0.5	4	−0.5	[65]
		1		−2.4	
		2		−14.5	
		4		−39.8	
		8		−74.2	
Comb White Leghorn	7-day-old	2.0	1	−11.0	[66]
			2	−12.0	
		4.0	1	−28	
			2	−31	
Leghorn	10-day-old	2.5	2	−27.5	[67]
		5		−28.0	
Petersen × Hubbard	1-day-old	2.5	1	−2.2	[68]
			2	−19.3	
			3	−22.7	
commercial broilers	1-day-old	2.0	5	−24.5	[69]
Plymouth Rock	2-week- old	1	2	−0.8	[59]
			4	−15.8	
			6	−18.5	
			10	−27.2	
		5	2	−31.5	
			4	−44.4	
			6	−60.9	
			10	−45.3	
Ross	1-day-old	0.4	1	−26.5	[70]
			2	−29.6	
			3	−41.1	
			4	−45.8	
			5	−48.7	
		0.8	1	−42.5	
			2	−47.7	
			3	−57.7	
			4	−63.7	
			5	−66.5	

Elaroussi *et al.* [70] reported that the body weight of chicks fed diets containing 0.4 and 0.8 mg/kg of OTA, from day one to five weeks of age, was one-third and one-half of that of the control group (OTA not added), respectively. Feed intake of chicks fed contaminated diets was lower than that of the control. Moreover, feed conversion rates of the groups fed contaminated diets at 0.4 and 0.8 mg/kg were, respectively, about 10 and 20% higher than the control. The mortality percentages during the experiment indicated a direct OTA dose dependent relationship. OTA intake significantly decreased thyroxine and hemoglobin concentrations, packed cell volume percentages, red and white blood cell counts, thymus weight, and humoral and cell-mediated immune responses in a dose and time dependent manner. 

OTA presence in feed is frequently associated with reduced egg production in laying hens. Verma *et al.* [71] studied the effect of different levels of OTA contamination in feed (1, 2 and 4 mg/kg) on protein and energy utilization in white Leghorn hens. Protein retention was negatively affected by OTA, suggesting a reduced nutrient absorption caused by the toxin. Energy partitioning showed a progressive decrease in egg deposition energy as OTA concentration increased. These alterations on protein and energy metabolism, together with the toxic effects previously described in poultry, may be responsible for the reduction of performance registered both in terms of number and weight of eggs produced by laying hens fed OTA.

The reduction of egg number and weight in hens exposed to several diets contaminated with OTA is summarized in Table 3. The reduction in the performance of laying hens was influenced by the level of contamination and length of exposure to a diet contaminated with OTA. In addition, temperature also had an effect: egg production was lower in laying hens reared at 33 °C than at 25 °C [72]. The reproductive performance of hens can also be negatively affected by diets highly contaminated with OTA. Indeed, Prior *et al.* [73] reported that eggs of Japanese quail females fed a diet with 16 mg/kg of OTA had significantly higher percentages of early embryonic deaths. Moreover, the same researchers, in a previous experiment comparing Japanese quail females fed either 0 or 16 mg/kg OTA for five weeks before mating and for the next four weeks, reported that fertility and hatchability of eggs were greatly reduced by the inclusion of OTA [74].

**Table 3:** Extent of reduction of eggs production and their weight in hens exposed to diets contaminated with OTA at different levels.

Breed	OTA in feed (mg/kg)	Exposition time	Change in number of eggs, in % respect to control *	Change in weight of eggs, in % respect to control *	Reference
Shaver 288	1.7	2 weeks	ns	ns	[72]
	3.1		−51.8	ns	
	3	(25 °C)	−24	ns	
	3	(33 °C)	−52.4	−3	
Hisex Brown	2	3 weeks	−6.9	−5.6	[108]
White leghorn	1	60 days	−21.6		[64]
Plymouth Rock	1	10 days	−10.8	−3.5	[80]
	5		−49.2	−19	

* Significant effects (p < 0.05) for all values, except where ns is reported.

#### 3.2.1. Interaction of OTA with Other Mycotoxins

A simultaneous contamination of poultry feedstuffs by OTA and other mycotoxins has been reported in several surveys [27,75].

The co-contamination by OTA and T-2 toxin resulted in additive toxic effects, causing a decrease in body weight, serum concentrations of total protein, and lactate dehydrogenase activity in poultry [76,77]. Moreover, Wang *et al.* [78] showed that OTA and T-2 combination impaired the immune function in broilers. Raju and Devegowda [69] reported negative additive effects on broilers body weight gain due to the addition of either aflatoxin B1 (3 mg/kg) or T-2 (3 mg/kg) to feed contaminated with OTA (2 mg/kg). Verma *et al.* [71] tested the effects of diets supplemented with OTA (1, 2 and 4 mg/kg), aflatoxin B1 (0.5, 1 and 2 mg/kg) and their combination on laying hens. The co-contamination produced synergistic adverse effects on protein retention, body and egg energy deposition and energy requirement for maintenance.

In broilers, the combination of OTA (2.5 mg/kg) and cyclopiazonic acid (34 mg/kg) increased the relative weight of the proventriculus, pancreas, kidney, and liver [68], enhancing the toxic effects of each single toxin.

#### 3.2.2. OTA Binders Supplements

Several feed supplements have been tested against the detrimental effects of OTA in the poultry industry. Stoev *et al.* [59] obtained a reduction of OTA toxic effect in chickens by adding aqueous extracts of Artichoke and Curcuma longa powder to feed. Similar results were reported in broilers by Sakhare *et al.* [79] with a polyherbal feed supplement. In chickens fed a diet contaminated with 5 mg/kg of OTA, the addition of 0.0025% of L-beta phenylalanine, 0.02% of Roxazyme-G, 8% of sesame seed in feed or water extract of artichoke at 5 ml/kg BW resulted in a reduction of intensity of macroscopical and histopathological changes, of organ weight modification, in an increase of body weight gain and in an improvement of hematological and biochemical parameters [59]. Among the products tested, the L-beta phenylalanine appeared to be the less efficient. The same experimental diets had positive effects on the egg production of laying hens [80]. When laying hens were fed diets contaminated by OTA, deoxynivalenol, nivalenol and T-2 toxin at 16, 218, 87 and 324 ppb, respectively, the inclusion of 0.125 g/kg of a synthetic antioxidant improved feed intake and egg production [81]. Moreover, the antioxidant was also able to reduce plasma uric acid and glucose concentrations in hens fed the contaminated diets. Positive effects on the performance of laying hens were also obtained by adding a commercial herbomineral (polyherbal extracts and hydrated aluminosilicates) toxin binder at 0.125% in feed artificially contaminated by OTA at 1 mg/kg [64]. 

Santin *et al.* [60] reported that the addition of 0.25% of aluminosilicate in diets of broilers receiving 2 mg/kg of OTA for 21 and 42 days did not avoid the macroscopic and microscopic alterations described in tissues of broilers exposed to OTA. Differently, the inclusion of a commercial product containing esterified-glucomannan (1 g/kg) in diets contaminated by OTA (2 mg/kg), aflatoxin B1 (3 mg/kg) and T-2 (3 mg/kg) was beneficial, increasing body weight gain, and decreasing the weight of liver and adrenals and the activity of GGT in chickens [69]. 

The evaluation of potential sequestering agents via *in vitro* experiments represents a powerful tool for screening their effectiveness in absorbing mycotoxins [82]. However, in some cases, there are significant discrepancies in the results between *in vivo* and *in vitro* experiments, regarding the effectiveness of the sequestering agent in reducing the absorption of the mycotoxins in the gastrointestinal tract. Garcia *et al.* [77] assessed two commercial sorbents for their ability to bind OTA and other mycotoxins *in vitro* and using chickens. The authors reported that the binding ability of sorbents observed *in vitro* was not confirmed in the *in vivo* experiment, in which the toxic effects of OTA in chickens could not be counteracted by the two sequestering agents tested.

In conclusion, experimental observations must be properly evaluated considering that the guidance values in the European Union countries for OTA in complementary and complete feedstuffs for poultry is 0.1 mg/kg (mg/kg), which is a much lower dose than that tested in many trials.

### 3.3. Cattle

The capability of cattle to degrade OTA has been demonstrated for many decades. Based on an *in vitro* experiment, Hult *et al.* [83] concluded that cows may degrade OTA in feeds contaminated with up to 12 mg/kg of this toxin. However, such ability is strictly related to rumen functionality. In fact Ribelin *et al.* [84] reported the death of calves within 24 h from an administration by a stomach tube of a single dose of 11 or 25 mg of OTA/kg of body weight. The relevance of rumen OTA degradation was clearly showed in two *in vivo* experiments on preruminant and functional ruminant calves [7]. In the first experiment, when OTA was administered at 1.0 or 4.0 mg/kg of BW to milk-fed (preruminant) calves, the animals died within the first 24 h after dosing, without differences between doses. In the second experiment, when OTA was administered at 2.0 mg/kg of body weight to calves with functional rumen fed a mixed diet of barley and hay, animals did not show any detectable negative effect. Moreover, authors showed that OTA present in serum of ruminant calves appeared to be absorbed relatively quickly and that OTA serum concentration–time curve had a bimodal disappearance trend, with a first higher peak observed at 2–4 h and a second at about 12.5 h after OTA intake, respectively. This bimodal trend in serum confirmed that the concentration of OTA in the bloodstream may be affected by the biliary excretion and intestine reabsorption process of OTA. 

Hydrolysis of OTA into ochratoxin-alpha and L-beta-phenylalanine has been observed in several *in vitro* experiments with cow rumen fluid [83,85]. In an *in vitro* trial, Müller *et al.* [86] showed that the OTA disappearance rate increased when grass silage was replaced by hay or when hay and grass silage were replaced by grass. Moreover the OTA disappearance was higher when the concentrate content passed from 10 to 50%. The authors suggested that these results may have been due to changes in the rumen protozoal population. In fact, the reduction of OTA is related to the increase of number and/or activity of protozoa, which is affected by the level of readily available carbohydrates in the diet. Indeed, Ozpinar *et al.* [87] indicated that the rate of OTA degradation increased together with the number of rumen protozoa and diet starch concentration. These results suggested that a high degradation rate of OTA could be reached by modulating rumen conditions in order to optimize the number and activity of protozoa. 

Ribelin *et al.* [84] indicated that the lethal single oral dose of OTA in cattle is probably higher than 13 mg/kg of body weight. Moreover, authors said that this amount of OTA could be hardly ever ingested in practice, also with the most contaminated grains. In fact, acute poisoning seems to be quite rare in cattle farms.

Therefore, in agreement with Youany and Diaz [88], the microbial activity in the gastrointestinal tract of cattle can be considered efficient in dramatically reducing OTA absorption and, consequently, it protects the animals against its toxic effects. Even though ochratoxicosis has been rarely reported in cattle, the accumulation of OTA in the bloodstream, as a consequence of chronic intake of contaminated feeds, should be prevented. In fact, long-term accumulation of OTA in tissues could represent a potential risk of its harmful effects in cattle. Therefore, also in cattle production, the best way of preventing the adverse effects of OTA is to minimize its intake.

### 3.4. Sheep and Goat

The toxic effects of OTA on goats have been demonstrated since the 1970s. Munro *et al.* [12] reported that intravenous infusion of OTA, at 1 mg/kg of body weight, caused the death of ewes within 24 hours. Ribelin *et al.* [84] reported that a goat treated with daily oral doses of OTA at 3 mg/kg of body weight, died on day five. In contrast, goats fed daily OTA at 2 and 1 mg/kg of body weight, for 14 days, did not show clinical signs of sickness or gross lesions in organs, whereas microscopic kidney changes were minimal. Moreover, serum glutamic-oxalacetic transaminase activity increased in association with the hepatocellular degenerative changes. 

Similarly to cattle, ochratoxicosis has been rarely reported in small ruminant species, such as sheep and goats, and the potential harmful effects of OTA are mainly related to the prolonged ingestion of a contaminated diet. This has led several researchers to study the degree of OTA detoxification in the digestive tract and the amount of OTA in the bloodstream of sheep. Xiao *et al.* [89] conducted a study both *in vitro* and *in vivo* to determine the extent of rumen OTA hydrolysis in sheep fed diets with different grain and hay ratios. OTA hydrolysis was not affected by the site of fluid sampling, *i.e.*, upper middle and lower sections of the rumen. An effect of the diet was observed in the *in vivo* experiment with hay-fed sheep showing higher rumen degradation than grain-fed sheep. Moreover, results of the *in vivo* trial confirmed those of the *in vitro* trial, indicating that higher OTA hydrolysis in hay-fed sheep was probably due to their larger rumen protozoa population. Because the rate of OTA hydrolysis decreased as rumen pH decreased, the authors suggested that when sheep are fed grains contaminated by OTA, detoxification may be effective by adding hay in the diet.

In a following study, Xiao *et al.* [90] evaluated the effects of diet on OTA bioavailability and hydrolysis in the rumen of sheep administered a single dose of toxin using a catheter implanted in the jugular vein (0.2 mg/kg of body weight) or a cannula inserted in the rumen (0.5 mg/kg of body weight). OTA concentration in serum was not detectable 120 h after its intravenous injection and showed a biexponential development. Moreover, from 56 to 61% of the OTA injected was excreted as OTA or Ochratoxin-alpha in the urine and feces. In particular, 90 to 97% of toxin administered was recovered as unchanged OTA in urine. The OTA concentration in serum of sheep treated with OTA injection in the rumen showed a biphasic disappearance, suggesting a kinetics with two open compartments in which the biliary recycling occurred. In those animals, about 90 to 99% of the OTA administered was excreted as Ochratoxin-alpha, whereas only 0.5–3% was excreted as unchanged toxin in urine. Moreover, in the experiment where the toxin was infused in the rumen, OTA hydrolysis in rumen was quicker in sheep fed hay than in sheep fed grain. The authors concluded that the rumen of sheep has a great aptitude for detoxifying OTA by hydrolysing it into Ochratoxin-alpha.

In wethers fed a diet with 70:30 concentrates to hay ratio for four weeks, Hohler *et al.* [91] evaluated effects of the addition of 2 and 5 mg/kg of OTA in the concentrate (*i.e.*, a daily intake of 22 and 55 mg of OTA/kg of body weight) on diet digestibility, concentrations of OTA and Ochratoxin-alpha in serum, and their excretion in urine and feces. OTA did not affect feed intake and body weight gain and no clear signs of sickness were observed. Moreover, nutrient digestibility did not differ between treated and control (OTA not added) groups. OTA serum concentration reached the highest value (about 10 ng/mL) at the end of the second week of treatment and remained stable throughout the experiment in wethers fed OTA at 2 mg/kg of concentrate. It was less stable (varying from 67 to 112 ng/mL) in serum of sheep fed OTA at 5 mg/kg of concentrate, in which the highest value was observed at the end of the experiment (day 27). In a preliminary trial, during which the concentrate was contaminated with OTA at 20 mg/kg, a visible intoxication and severe reduction of feed intake of wethers was demonstrated. Moreover, after two weeks, the OTA serum concentration was on average 36 ng/mL, with a high variability between animals. In that case, the daily dose of OTA of 225 mg/kg of body weight caused clinical signs very close to those of acute ochratoxicosis. The Ochratoxin-alpha excreted in urine and feces was greater than 75% of the OTA ingested, and the amount found in urine was about 60 to 70% of the total. Hohler *et al.* [91] concluded that the actual ability of sheep to degrade OTA in the rumen is lower than that observed in (other) experiments in which OTA was artificially added in the diet or injected in the rumen. 

Blank *et al.* [92] studied OTA metabolism in sheep fed diets diet with 70:30 concentrates to grass silage ratio to which contaminated wheat was added, to obtain daily dosages of 0.387, 0.774, and 1.161 mg of OTA/head for a period of 29 days. The conversion of OTA in Ochratoxin-alpha was partial and much lower than that reported in previous *in vivo* and *in vitro* trials. Moreover, the transfer of OTA into the bloodstream was linearly and positively related with its ingestion, and OTA was detected also in the serum of sheep fed less than 4.4 mg of OTA/day. These data confirm that the concentration of OTA in serum is higher (six- to nine-fold) than that of Ochratoxin-alpha, although no sheep showed health disorders and the feed offered was always consumed completely. Those results clearly indicate that also in small ruminants the amount of OTA from contaminated diets that reaches the bloodstream is relevant and, therefore, the carry over of OTA into animal products must be taken into consideration. 

Recently, Blank and Wolffram [93] investigated whether a daily addition in the diet of 0.4 g of live yeast cells (*Saccharomyces cerevisiae*), registered as a feed additive for improving livestock performance, could reduce the OTA bioavailability and enhance its excretion in sheep. The authors studied the metabolism of OTA in castrated male sheep (about 89 kg of body weight) after administration of a single dose of OTA (2.46 mg) as naturally contaminated wheat. The addition of live yeast cells did not affect OTA toxicokinetics and toxicodynamics. Moreover, the conversion of OTA into Ochratoxin-alpha was incomplete and lower than that reported in experiments *in vitro*.

The terminal excretion half-life of OTA from blood is short (about 16 h) indicating that in sheep the binding of OTA with serum albumin is lower than in other livestock species such as cattle or pigs.

It remains unclear whether the continuous administration of OTA contaminated diets, even at a low level, affects sheep and goats’ health as a consequence of toxin accumulation in the different organs involved in its detoxification or elimination processes.

OTA transfer from diet in to milk in lactating small ruminants was studied by Nip and Chu [94] by monitoring the distribution of a single dose (0.5 mg/kg of body weight) of [3H]-label-OTA in milk, urine, feces and serum blood of goats for seven days. The distribution of total radioactivity in feces, urine, milk and serum was 54, 38, 6 and 2.26%, respectively. The results showed that only 0.026% of the OTA administered was found in milk during the seven-day period.

## 4. OTA Presence in Meat, Eggs and Milk

OTA ingested from contaminated diet by livestock species reaches the bloodstream where it persists for long time and may accumulate in organs responsible for its detoxification and excretion. Therefore, it is important to verify the carry-over of this mycotoxin into animal products such as meat, eggs and milk.

Transfer of OTA along the food chain of animal products depends essentially on the following factors: the extent of exposure of animals to an OTA-contaminated diet; the level of transfer of intact OTA into the bloodstream of animals; the degree of OTA persistency in the blood and its accumulation in different tissues; and the magnitude of the transfer of OTA from blood to milk, meat or eggs.

Human exposure to intake of OTA is mainly due to the consumption of contaminated cereals, however an indirect source of OTA exposure may be the consumption of products derived from animals fed contaminated diet. Moreover, the exposure of lactating women to food indirectly contaminated by OTA, such as animal products, may represent a potential way of transferring this toxin to newborn suckling children by milk. This was suggested in a survey carried out on Norway by Skaug *et al.* [95] indicating that women with a high dietary intake of liver paste were more likely to have OTA contaminated milk.

In a review, Jørgensen [96] reported a historical view (from 1969 until July 2005) of the number of publications regarding the different commodities contaminated by OTA. About 19% of articles focused on OTA in meat and meat products, whereas no publications dealt with OTA in other animal products, such as milk or eggs.

Because OTA has high affinity to blood proteins, in particular to serum albumin, this toxin can likely accumulate in different organs of animals used for food such as muscle and liver.

### 4.1. Swine

Since the 1970s, the presence of OTA in muscle, fat, liver and kidneys of pigs fed diets contaminated with OTA has been documented. In fact, Krogh *et al.* [97] showed that OTA concentration differed among pig tissues, with increasing concentrations of OTA being observed from fat, muscle, liver, to kidney. Moreover, authors found that in all tissues (kidney, liver, muscle and fat) the disappearance of OTA after termination of exposure to contaminated diet, showed an exponential trend and values of half-life of residues ranged from three to five days.

In several experiments in which pigs were fed diets with different OTA content for different times, the toxin was detected in different tissues. For example, Jarczyk *et al.* [44] reported that OTA concentrations in kidney, liver and longissimus dorsi muscle (8.74, 5.90 and 4.26 ppb, respectively) of gilts were lower than those of the diet supplied (32.2 ppb of OTA for 14 days). Similar results were reported by Aoudia *et al.* [45] that found OTA concentrations in kidney and liver of 12.49 and 1.02 ppb, respectively in four-week-old piglets fed a contaminated diet with 117.45 ppb of OTA for 28 days. In an experiment carried out on adult pigs, Dall’Asta *et al.* [98] showed that the administration of diets contaminated by OTA at 200 ppb for 40 days led to an average OTA content of 2.21 ppb in samples of raw ham muscle.

In contrast, Malagutti *et al.* [43] reported OTA concentrations in piglet kidney, liver and semimembranous muscle of 69, 52 and 6.1 ng/g, respectively, after four months of administration of a contaminated diet at the dose of 25 ppb. In this case, OTA concentration in the diet was lower than in kidney and liver, but higher than in muscle.

These contrasting results could be mainly explained by differences in the duration of exposure of animals to contaminated diets, which is positively related to OTA in blood. This explanation is supported by the study of Stoev *et al.* [42], in which the concentration of OTA in serum was higher in pigs fed OTA contaminated diet for six months than in those fed the same diet for three months.

The content of OTA in kidney and liver appears to be affected by the time occurring between the last ingestion of contaminated diet and the slaughter of animals. Jarczyk *et al.* [44] showed that the average OTA content of blood, liver and kidney tended to be higher in pigs which had been fed for the last time five hours before slaughter than in those fed 18 hours before slaughter.

The highest OTA concentration is normally found in kidneys, mainly due to the higher blood flow/mass ratio in these organs compared to others such as liver or muscle. In addition, OTA is reabsorbed in all nephron segments and this fact delays its elimination, thus increasing the risk of OTA accumulation in kidney tissue [4].

Since the 1970s, several studies have established the quantitative relationships between concentrations of OTA in various tissues of pigs [37,38,40]. Based on this experimental evidence, Denmark established guidelines to control OTA levels in pork products in their slaughter houses. These guidelines determine that in the case of macroscopic changes, pig kidneys have to be analyzed for their OTA content and: (i) if the OTA in kidney is higher than 25 mg/kg, the whole carcass is rejected, because the meat is supposed to be highly contaminated; (ii) if the OTA in kidney is between 10 and 25 ppb, edible offals are eliminated; and (iii) if the OTA in kidney tissue is lower than 10 ppb, only the kidneys are discarded [99]. 

Other European countries have also set guidelines to control the presence of OTA in edible pork tissues, such as Italy (1 ppb for pig meat and derived products, as maximum concentration allowed) and Estonia (10 ppb for pig liver) (FAO, 2004). Other countries, such as France, have done so in the past and are now developing monitoring plans of OTA occurrence in pig kidneys as a tool to evaluate the risk assessment for pork tissue consumers [100].

During the last years, several surveys have been carried out in many European countries on OTA in blood and/or in edible tissues of pigs. The results are summarized in Table 4. 

A wide variation in the incidence of positive samples was observed. These results should be carefully evaluated by considering the limit of detection (LOD) and limit of quantification (LOQ) of the analytical methods used. Moreover, all authors of the surveys cited above suggested that the actual OTA concentration in pork tissues is generally lower than in other food sources and may not represent a health hazard for consumers. Despite that, all authors proposed the extension of the control system based on sampling pork tissues because it is important to keep OTA levels in pork products and, therefore, human OTA consumption as low as possible.

Ferrufino-Guardia *et al.* reported the effective transfer of OTA from blood to milk in lactating rabbits, another monogastric livestock species, as a consequence of intake of contaminated diet (10–20 g/kg of body weight per day) [107]. This study showed that OTA concentration in milk was positively related with its concentration in plasma and its ingested amounts. The high correlation of OTA in milk and in plasma suggests that the passage of this toxin from the bloodstream into the milk is by passive diffusion. In addition, this study showed that OTA accumulates in the kidney, liver, mammary gland and muscle of rabbit, as reported in other animal species.

**Table 4:** Incidence of OTA in tissues of slaughtered pigs in different countries.

Tissue	No. samples	LOD (ppt)	LOQ (ppt)	Incidence of positives (%)	Mean of positives (ppt)	Range (ppt)	Country	Reference
kidney	300	0.05–0.2	0.15–0.5	13.4	0.81	>0.17–1.4	France	[101]
kidney	710	0.11–0.2	0.5	25.9		0.5–5	France	
kidney	300	0.02	0.06	94.7	0.5	0.02–15	Denmark	[102]
muscle fillet	300	0.03	0.09	76	0.12	0.03–2.9	Denmark	
muscle for ham	22	0.01	0.03	9	0.05	0.04–0.06	Italy	[103]
kidney	54	0.01	0.52	100	0.29		Italy	[104]
muscle	54	0.01		77.8	0.024		Italy	
kidney	90	0.01		33.3	1.26	0.17–52.5	Serbia	[105]
liver	90	0.01		26.6	0.63	0.22–14.5	Serbia	
kidney	60	0.5	1	70	3.97	1.3–22.0	Serbia	[106]
liver	60	0.5	1	65	3.2	1.2–19.5	Serbia	

### 4.2. Poultry

Bozzo *et al.* [63] analyzed the OTA content in the liver, kidney, and muscles of 10 layer hens that received feed contaminated with OTA (ranging between 160 and 332 ppt) for at least two months in two farms. The highest OTA level was found in the kidneys (on average 13.65 ± 3.58 ppt, range: 8.7 to 16.9 ppt), whereas in liver it averaged 4.43 ± 0.64 ppt (range 3.7 to 5.1 ppt). OTA was not detected in muscles (LOD = 0.10 ppt). Similarly, in laying hens fed a diet contaminated by OTA at 2 mg/kg of feed for three weeks, Denli *et al.* [108] found 15.1 μg/kg of OTA in the liver, but did not detect (LOD = 0.15 ppt) OTA residues in eggs. 

When hens were fed diet containing OTA at 0.3 and 1 mg /kg of feed, Krogh *et al.* [109] did not detect OTA in eggs. In contrast, Juszkiewicz *et al.* [110] detected OTA in the eggs of laying hens fed OTA at 10 mg/kg of body weight. These results suggest that the passage of OTA from fed into eggs is possible, but only when OTA intake is very high. Therefore, the risk of OTA intake by humans as a consequence of eggs consumption is extremely low.

Also in poultry, Biró *et al.* [63] reported that the residues of OTA accumulated in all organs, with high levels in liver and kidneys and low levels in muscle. In particular, in chickens fed 0.5 mg of OTA per animal weekly for four weeks, after the first two weeks of exposure, OTA was found at 0.28 and 0.20 ng/g in breast and thigh muscle, respectively. Then, OTA residue in muscle increased slightly, reaching its maximum value after four weeks, when the concentration of OTA was 0.84 ng/g in both white and red muscles. 

So far, scientists seem not to be really interested in the study of OTA contamination occurrence in poultry. Probably this is mainly because in field conditions it is highly improbable that OTA contamination of poultry diets is high enough to cause residue accumulation of this toxin in meat. 

However, since OTA in poultry tends to concentrate mainly in the liver, which is used for producing the Foie Gras in ducks and geese, it would be advisable to implement monitoring programs on OTA occurrence also on poultry edible tissues, as recently adopted in France [100].

### 4.3. Ruminants

In several ruminant species, such as cattle, ovine and caprine, OTA transfer in meat and milk as a consequence of contaminated feed ingestion is a rather infrequent event in field conditions, being the toxin processed by rumen microorganisms into less toxic metabolites which are mainly excreted in urine and feces [57].

However, under experimental conditions, transfer of OTA administered *per os* in cow [84] and goat [94] milk has been reported since the 1970s. Ribelin *et al.* [84] showed that a cow given a single dose of OTA at 13.3 mg/kg of body weight, excreted 4.5 mg of OTA in milk during following day, but the toxin was no longer detected in the milk produced afterwards. On the other hand, in cows given OTA 1.66 mg/kg of body weight/d for four days, traces of toxin were detected in milk on days three, four and five; however, for doses of 0.2 or 0.75 mg/kg of body weight, OTA was never detected in milk during the experiment.

Nip and Chu [94] showed that when two lactating goats were given a single oral dose of 3H-OTA (0.5 mg/kg of body weight), only 0.026% of the toxin was found in milk during the following seven days.

A further confirm of the low transfer of OTA from diet into meat was reported by Shreeve *et al.* [111]. In particular, when two adult cows were administered concentrate diets containing 0.317–1.125 mg of OTA/kg for 11 weeks before slaughter, no residues of OTA were detected in muscle, liver, kidney, serum, milk or urine. Just for one cow, OTA residues (about 5 ppt) were found in kidneys. 

During the last decades, other studies have been conducted to confirm the ability of ruminants to degrade OTA, to reduce its passage into the bloodstream and to minimize toxic effects in the food chain.

Xiao *et al.* [90] confirmed an efficient ruminal OTA hydrolysis in sheep in a trial carried out on four female Suffolk sheep given a single dose of OTA (0.5 mg/kg of body weight). Moreover, the amount of OTA reaching the bloodstream was much lower in hay-fed than in grain-fed sheep.

OTA bioavailability in the bloodstream of sheep was confirmed by Höhler *et al.* [91]. Wethers fed 2 mg of OTA/kg feed for four weeks showed a constant blood content of the toxin (about 10 ng/mL) throughout the trial. For a higher level of contamination (5 mg of OTA/kg of feed), the concentration of the toxin in blood increased markedly together with a higher variability (67 to 112 ng/mL of serum).

Even if the transfer of OTA from feed into milk of lactating ruminants has not been clearly demonstrated, few surveys have been carried out to verify the occurrence of OTA in cow milk. Valenta and Goll [112], analyzing 121 samples of cow milk collected in northern Germany (LOD = 0.01 ng/mL), did not find OTA in all analyzed samples. In contrast, other authors reported the occurrence of OTA in cow milk. For example, Breitholtz-Emanuelsson *et al.* [113] found that five out of 36 cow milk samples collected in Sweden were contaminated by OTA (range of 10–40 ng/L). Similarly, in a survey on Norwegian conventional and organic dairy cow farms, Skaug [114] detected OTA in six out of 40 conventional (ranging from 11 to 58 ng/L), and in five out of 47 organic (ranging from 15 to 28 ng/L) milk samples, respectively. Moreover, no statistically significant differences were found in OTA content between milk samples produced in organic or conventional farms. More recently, Boudra *et al.* [115] studied OTA occurrence in milk produced in French dairy cow herds during 2003 summer and winter 2003. The toxin was detected in three out of 264 samples, at low levels (5.0, 6.0 and 6.6 ng/L).

Low frequency of OTA occurrence and concentration reported in the above cited studies suggest that, even if milk can be a possible source of this toxin for human diet, it does not represent a particular risk for adult consumers. However, OTA presence in milk may represent a danger for some categories such as children, who consume large amounts of milk.

## 5. Conclusions

Ocratoxicosis may occur in farms where feeds are contaminated by OTA. The risk of contamination is high for cereals and other grains, depending on environmental conditions during crop production and storage, whereas it is low in all kind of forages.

OTA contaminated feed affects primarily animal metabolism and health. Under field conditions, less intense effects have been reported for productive and reproductive performances and on product quality and safety. Among livestock, monogastric species are more susceptible to OTA than poligastric ones, due to the combined effect of increased exposure to contaminated feeds and the lack of detoxification by the rumen.

Pigs show a significant and linear reduction of daily gain with increasing doses of ingested OTA. In this species, OTA tends to accumulate in kidneys and, to a lesser extent, in the liver. This may represent a potential danger for human food chain. Indeed, the occurrence of OTA in fat and muscle has been extensively documented as well. However, observed toxin concentrations in pork edible tissues are generally low compared with other food sources and does not represent a health hazard for consumers.

Poultry are less sensitive to OTA than pigs because of their higher efficiency in excreting the toxin. Nevertheless, several experiments show a reduction in performance mainly due to the negative action of the toxin in various organs and metabolic pathways. In broilers, as concentration of toxin in the feed and time of exposure increase, daily growth decreases. Similarly, laying hens reduce eggs weight and production as OTA concentration in feed and time of exposure increase. Poultry accumulate OTA in meat and in eggs in relevant amounts only if exposed to high levels of feed contamination, rather unlikely to find under field conditions. An exception might be represented by food like the French Fois Gras, because OTA tends to accumulate in liver at concentrations even higher than those in feeds given to ducks or geese. 

Cattle, sheep and goats are able to degrade OTA in rumen mainly through the action of protozoa thus acute poisoning seems to be an infrequent event under field conditions. Rumen functionality represents the main barrier to the passage of toxin into the bloodstream, so that the correct feeding of animals is the main preventive action against ocratoxicosis. Even if ruminants ingest OTA contaminated feed, the concentration of OTA in their main products remains either below the limits of detection/quantification or under the threshold of risk for consumer health.

In conclusion, feed contamination with OTA is a problem almost exclusively of monogastric livestock, mainly due to the economic losses caused by the reduction of production performances, to the negative impact on animal health and welfare, and to the possibility of toxin transfer into edible tissues of intoxicated animals
